# Blood neutrophil-lymphocyte ratio predicts survival for stages III-IV gastric cancer treated with neoadjuvant chemotherapy

**DOI:** 10.1186/1477-7819-11-112

**Published:** 2013-05-24

**Authors:** Hailong Jin, Geer Zhang, Xiaosun Liu, Xiaokun Liu, Chao Chen, Hang Yu, Xiaomei Huang, Qing Zhang, Jiren Yu

**Affiliations:** 1 Department of Gastrointestinal Surgery, the First Affiliated Hospital, Medical College, Zhejiang University, No. 79, Qingchun Road, Hangzhou, Zhejiang Province 310003, China

**Keywords:** Gastric cancer, Neutrophil-lymphocyte ratio, Univariate analysis, Multivariate analysis, Prognosis

## Abstract

**Background:**

Accurate predictors of survival for patients with advanced gastric cancer treated with neoadjuvant chemotherapy are currently lacking. In this study, we aimed to evaluate the prognostic significance of the neutrophil-lymphocyte ratio (NLR) in patients with stage III-IV gastric cancer who received neoadjuvant chemotherapy.

**Methods:**

We enrolled 46 patients in this study. The NLR was divided into two groups: high (>2.5) and low (≤2.5). Univariate analysis on progression-free survival (PFS) and overall survival(OS) was performed using the Kaplan-Meier and log-rank tests, and multivariate analysis was conducted using the Cox proportional hazards regression model. We analyzed whether chemotherapy normalized high NLR or not, and evaluated the prognostic significance of normalization on survival.

**Results:**

The univariate analysis showed that PFS and OS were both worse for patients with high NLR than for those with low NLR before chemotherapy (median PFS 16 and 49 months, respectively, *P* = 0.012; median OS 21 and 52 months, *P* = 0.113). PFS and OS were also worse for patients with high NLR than for those with low NLR before surgery (median PFS 12 and 35 months, *P* = 0.019; median OS 21 and 52 months, *P* = 0.082). Multivariate analysis showed that both NLR before chemotherapy and surgery were independent prognostic factors of PFS. Neoadjuvant chemotherapy normalized high NLR in 11 of 24 patients, and these 11 patients had better median PFS and OS than the 13 patients who had high NLR both before chemotherapy and before surgery (PFS: 35.0 and 10.0 months, *P* = 0.003; OS: 60 and 16 months, *P* = 0.042).

**Conclusions:**

NLR may serve as a potential biomarker for survival prognosis in patients with stage III-IV gastric cancer receiving neoadjuvant chemotherapy.

## Background

Gastric cancer is the one of the most common cancer types and is the leading cause of cancer-related death worldwide. Annually, nearly 1 million new cases are diagnosed, and more than 700,000 deaths are estimated to occur from this disease [1]. Increased use of adjuvant and neoadjuvant treatment regimens such as radiotherapy and neoadjuvant chemotherapy has greatly improved both progression-free survival (PFS) and overall survival (OS) [2], and it has been reported that it is possible to deliver neoadjuvant chemotherapy without increasing surgical morbidity and mortality compared with surgery alone [3]. However, it is necessary to find accurate predictors of outcomes for neoadjuvant chemotherapy in order to identify those patients who are more likely to benefit from neoadjuvant chemotherapy. Although a few serum biomarkers have been found to be associated with poor prognosis in patients with gastric cancer [4,5], their use is often time-consuming and expensive. Recently, novel immunological and histological biomarkers have been identified [6-8]. However, these largely depend on specimens obtained after resection of the primary tumor, and this limits their use in clinical practice prior to surgery.

A systemic inflammatory response has been reported to be associated with the progression of cancer [9,10]. Coussens *et al*. [9] reported that the ability of a tumor to invade and metastasize was dependent on the intrinsic characteristics of the tumor cells, as well as the tumor microenvironment. Peripheral blood tests at the time of diagnosis and treatment can reflect inflammatory conditions within the tumor. Evaluation of peripheral blood parameters including C-reactive protein (CRP), leukocytes, neutrophil, lymphocyte, monocyte, and platelet counts, as well as the modified Glasgow Prognostic Score (mGPS), neutrophil to lymphocyte ratio (NLR), and platelet to lymphocyte ratio (PLR), have been proposed as prognostic factors for patients with various types of malignancies. An elevated serum CRP level was associated with poor survival in patients with gastro-esophageal cancer [11], ovarian cancer [12], and renal cancer [13], while elevated neutrophil, monocyte, and leukocyte counts have been reported to be associated with poor survival in patients with metastatic melanoma [14,15]. A high pre-operative NLR has been identified as a useful and convenient predictor of survival in patients with gastric cancer [16-20], colorectal cancer [21,22] and advanced non-small-cell lung cancer [23]. Recently, Dutta S *et al*. [24] reported that the mGPS was independently associated with cancer-specific survival in patients undergoing potentially curative resection of gastric cancer, and an increase in the mGPS was associated with higher NLR and poorer survival. However, the usefulness of NLR in patients with advanced malignancies receiving neoadjuvant chemotherapy has been reported only rarely [25-27]. Kunisaki C *et al*. [25] found that there were significant correlations between the GPS and NLR in patients with advanced gastric cancer receiving biweekly docetaxel and S-1 combination chemotherapy, and patients with low GPS group may obtain favorable outcomes with chemotherapy. An analysis conducted by Kishi *et al*. [26] showed that a high NLR independently predicted poor survival in patients with colorectal liver metastases treated with chemotherapy followed by resection or chemotherapy alone, and that normalization of the high NLR by neoadjuvant chemotherapy indicated improved survival.

To our knowledge, the prognostic significance of pre-treatment NLR including pre-chemotherapy and pre-operative NLR have not been evaluated in advanced gastric cancer treated with neoadjuvant chemotherapy. The aim of the present study was to evaluate the prognostic significance of pre-chemotherapy and pre-operative NLR in peripheral blood samples from patients with stage III-IV gastric cancer receiving neoadjuvant chemotherapy. In addition, we determined whether neoadjuvant chemotherapy normalized high pre-chemotherapy NLR, and assessed the effect of NLR normalization on survival.

## Methods

### Ethics approval

The study was approved by the ethics committee of the First Affiliated Hospital, Medical College, Zhejiang University, and prior to the research, informed consent was obtained from all patients.

### Patients

In total, 58 patients diagnosed with advanced gastric cancer and treated with neoadjuvant chemotherapy in the Department of Gastrointestinal Surgery, First Affiliated Hospital, Medical College, Zhejiang University were enrolled in this study between July 2004 and May 2009. All patients fulfilled the following criteria: locally advanced, T3-T4 carcinoma staged according to the sixth edition of the American Joint Committee on Cancer (AJCC) guidelines, without distant metastases; no previous chemotherapy or radiotherapy; and adequate hematological, liver and renal function. Of the 58 patients, 3 had not undergone gastrectomy (2 had undergone opening and closure, and one had undergone palliative gastrojejunostomy), 3 had been diagnosed with other malignancies simultaneously (2 with rectal cancer and 1 with lung cancer), and another 2 patients showed evidence of infection at the time of blood sampling, thus ultimately, 50 patients were eligible for inclusion in this study.

**Figure 1:**
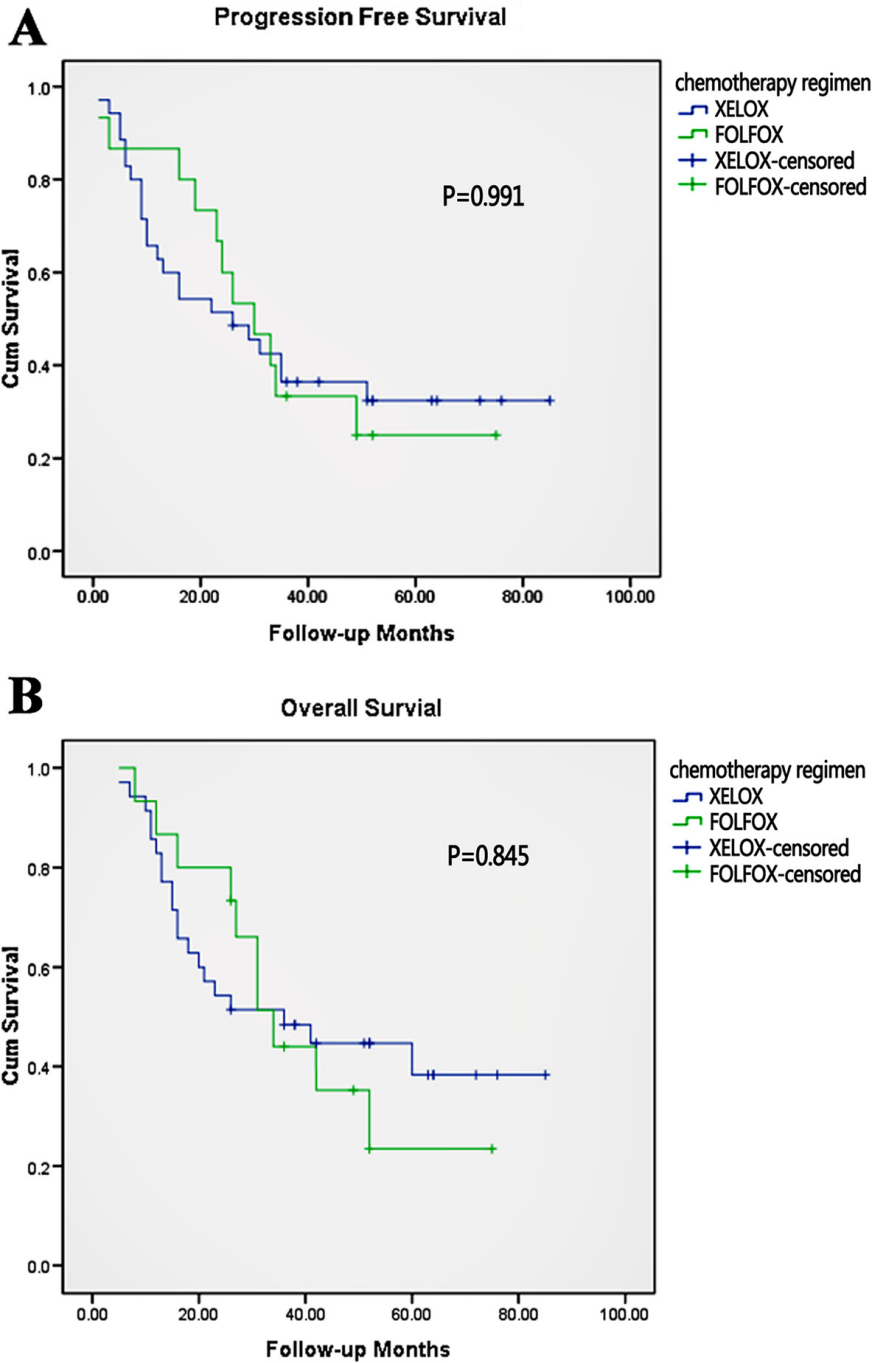
**Survival of patients treated with different neoadjuvant chemotherapy regimens.** (**A**) Progression-free survival and (**B**) overall survival.

Patients were treated with one of two neoadjuvant chemotherapy regimens: oxaliplatin plus capecitabine (XELOX) or oxaliplatin plus 5-fluorouracil (5-FU) plus leucovorin (FOLFOX). As there were no significant differences between the two chemotherapy regimen groups regarding either median PFS (26 versus 30 months; *P* = 0.991; Figure 1A) or median OS (36 versus 34 months; *P* = 0.845; Figure 1B), data from both groups were combined in this study. For 4 of the 50 eligible patients, data on blood parameters before surgery were not available (missing data <10%). Thus, data from 46 patients were analyzed in the present study.

### Blood parameters

Venous blood samples were taken at the time of diagnosis before neoadjuvant chemotherapy, and 4 weeks or more after the last dose of chemotherapy and within 1 week before surgery (when hematotoxicity had been minimized). NLR was defined as neutrophil count divided by lymphocyte count. The cut-off values for white blood cells (>6,000/mm^3^ and ≤6,000/mm^3^), neutrophils (>4,000/mm^3^ and ≤4,000/mm^3^), lymphocytes (>1,500/mm^3^ and ≤1,500/mm^3^), monocytes (>500/mm^3^ and ≤500/mm^3^) and NLR (>2.5 and ≤2.5) were defined using the median values and data from previous studies [17].

### Statistical analysis

Response rates were evaluated according to the Response Evaluation Criteria In Solid Tumors (RECIST) guidelines [28]. Clinical response was defined as either complete response (CR) or partial response (PR), and non-response as either stable disease (SD) or progressive disease (PD). Clinical benefit was defined as CR, PR or SD, and no benefit was defined as PD. The follow-up period commenced at the start of neoadjuvant chemotherapy with a censor date of April 2012. PFS was calculated from the date of initiation of neoadjuvant chemotherapy until objective tumor progression, death, or last contact. OS was calculated from the date of initiation of neoadjuvant chemotherapy until death or last contact. Potential prognostic factors were age, gender, tumor site, clinical response, clinical benefit, type of surgery, radicality of surgery, tumor differentiation, and peripheral blood parameters; these were entered into a univariate analysis using the Kaplan–Meier analysis model and differences between groups were compared by log-rank test. Prognostic factors with significance values of *P*<0.10 in the univariate analysis were entered into a multivariate analysis, which was performed using Cox proportional hazards model with the backward likelihood method to test for independent prognostic variables.

All statistical analyses were performing using SPSS software (version 16.0; SPSS Inc., Chicago, IL, USA), and in all analyses, a value of *P* <0.05 was considered to indicate statistical significance.

## Results

### Patient characteristics

Table 1 shows the characteristics of the 46 patients: 36 were male and 10 were female, with a median age of 60 years (range 37–77 years). Of the 46 patients, 32 received a XELOX neoadjuvant chemotherapy regimen and 14 patients received a FOLFOX regimen. The median number of chemotherapy cycles was three (range one to five). All 46 patients underwent gastrectomy; 28 (60.9%) underwent total gastrectomy and 18 (39.1%), subtotal gastrectomy, with 1 patient receiving combined resection of the transverse colon. Clinical and pathological TNM (tumor, node, metastasis) classification based on the AJCC staging were as follows: clinical TNM classification showed that 40 patients had stage III disease and 6 had stage IV disease, while pathological TNM classification based on specimens obtained after resection of the primary tumor showed that 23 patients had stage III disease and 11 had stage IV disease.

**Table 1:** Demographic and clinicopathological characteristics of 46 patients with gastric cancer

**Parameter**	**Number (%)**
Age, years	
>65	14 (30.4%)
≤65	32 (69.6%)
Gender	
Male	36 (78.3%)
Female	10 (21.7%)
Chemotherapy regimen	
XELOX	32 (69.6%)
FOLFOX	14 (30.4%)
Cycles of chemotherapy, median (range)	3 (1–5)
Primary tumor site	
Upper 1/3	12 (26.1%)
Middle 1/3	12 (26.1%)
Low 1/3	22 (47.8%)
Clinical response^a^	
Yes	21 (45.7%)
No	25 (54.3%)
Clinical benefit^b^	
Yes	43 (93.5%)
No	3 (6.5%)
Types of surgery	
Total	28 (60.9%)
Subtotal	18 (39.1%)
Radicality	
R0	37 (80.4%)
R1	0
R2	9 (19.6%)
Combined resection	
No	45 (97.8%)
Yes	1 (2.2%)
Differentiation	
Differentiated^c^	15 (32.6%)
Poorly differentiated^d^	31 (67.4%)
Clinical TNM classification^e^	
T stage	
T3	40 (87%)
T4	6 (13%)
N stage	
N1	14 (30.4%)
N2	32 (69.6%)
N3	0
TNM stage	
III	40 (87%)
IV	6 (13%)
Pathological TNM classification^e^	
y T stage^f^	
T0	2 (4.3%)
T1	1 (2.2%)
T2	6 (13%)
T3	28 (60.9%)
T4	9 (19.6%)
y N stage^f^	
N0	8 (17.4%)
N1	19 (41.3%)
N2	13 (28.3%)
N3	6 (13%)
y TNM stage^f^	
Tis	1 (2.2%)
I	3 (6.5%)
II	8 (17.4%)
III	23 (50%)
IV	11 (23.9%)

CR, complete response, PD, progressive disease; PR, partial response; SD, stable disease; Tis, tumor *in situ*; TNM, tumor, node metastasis;

^a^Clinical response: Yes means CR and PR; No means SD and PD.

^b^Clinical benefit: Yes means CR and PR and SD; No means PD.

^c^Well and moderately differentiated adenocarcinoma.

^d^Poorly differentiated adenocarcinoma, ring cell carcinoma.

^e^American Joint of Committee On Cancer, sixth edition.

^f^Classification after neoadjuvant chemotherapy.

### Blood parameters

The median pre-chemotherapy white blood cell, neutrophil, lymphocyte, and monocyte counts were 6,400, 3,900, 1,550, and 500 per mm^3^, respectively. The median pre-chemotherapy NLR was 2.74 (range 0.91 to 7.00), and the median pre-operative NLR was 2.35 (range 0.70 to 6.00). An NLR value of 2.5 was used as the cut-off value to classify patients into high (>2.5) or low (≤2.5) NLR groups.

### Prognostic variables for PFS and OS

**Figure 2:**
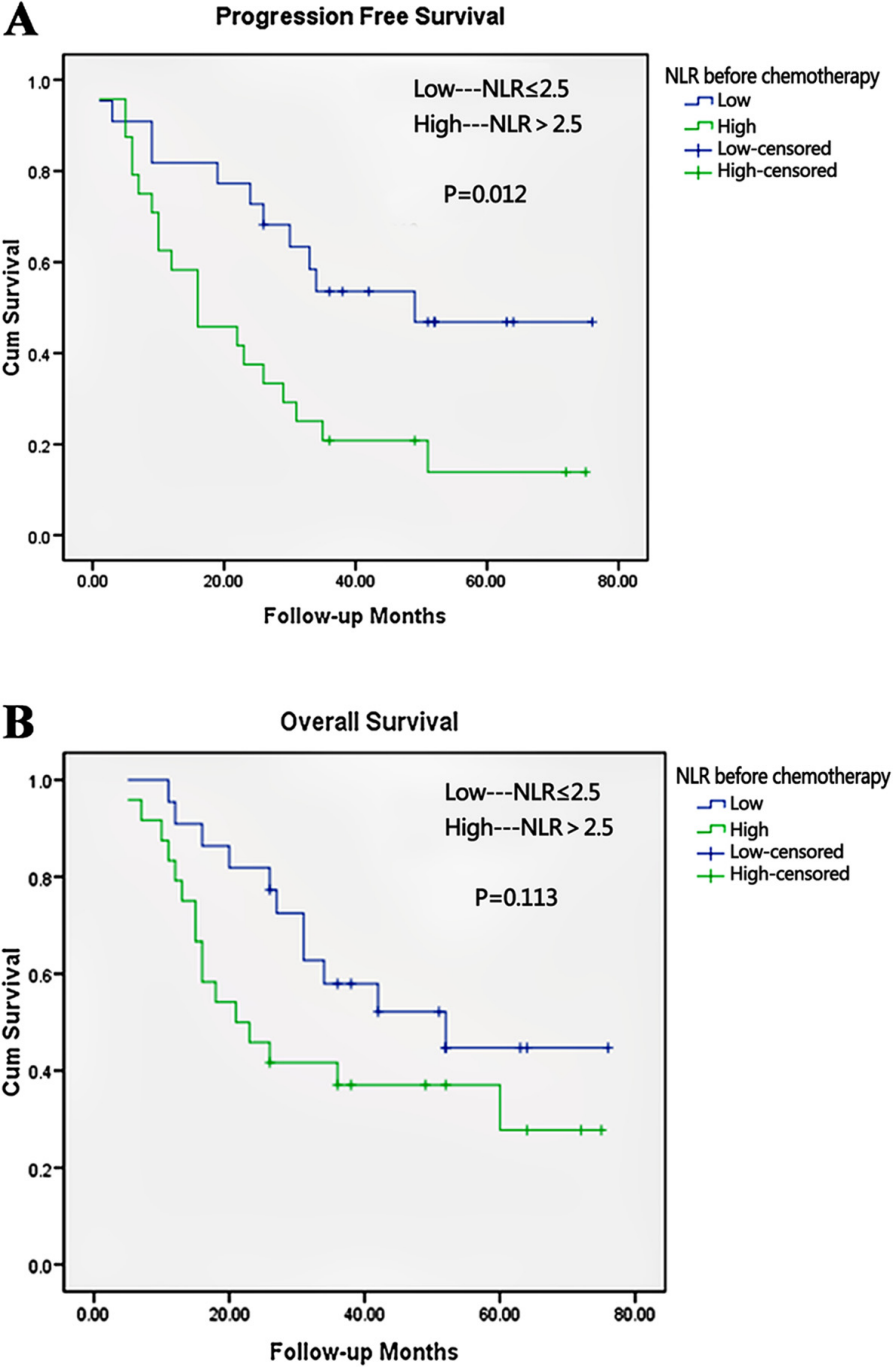
**Kaplan-Meier survival curves according to neutrophil-lymphocyte ratio (NLR) before neoadjuvant chemotherapy.** (**A**) Progression-free survival and (**B**) overall survival. *P* values were determined using the log-rank test.

**Figure 3:**
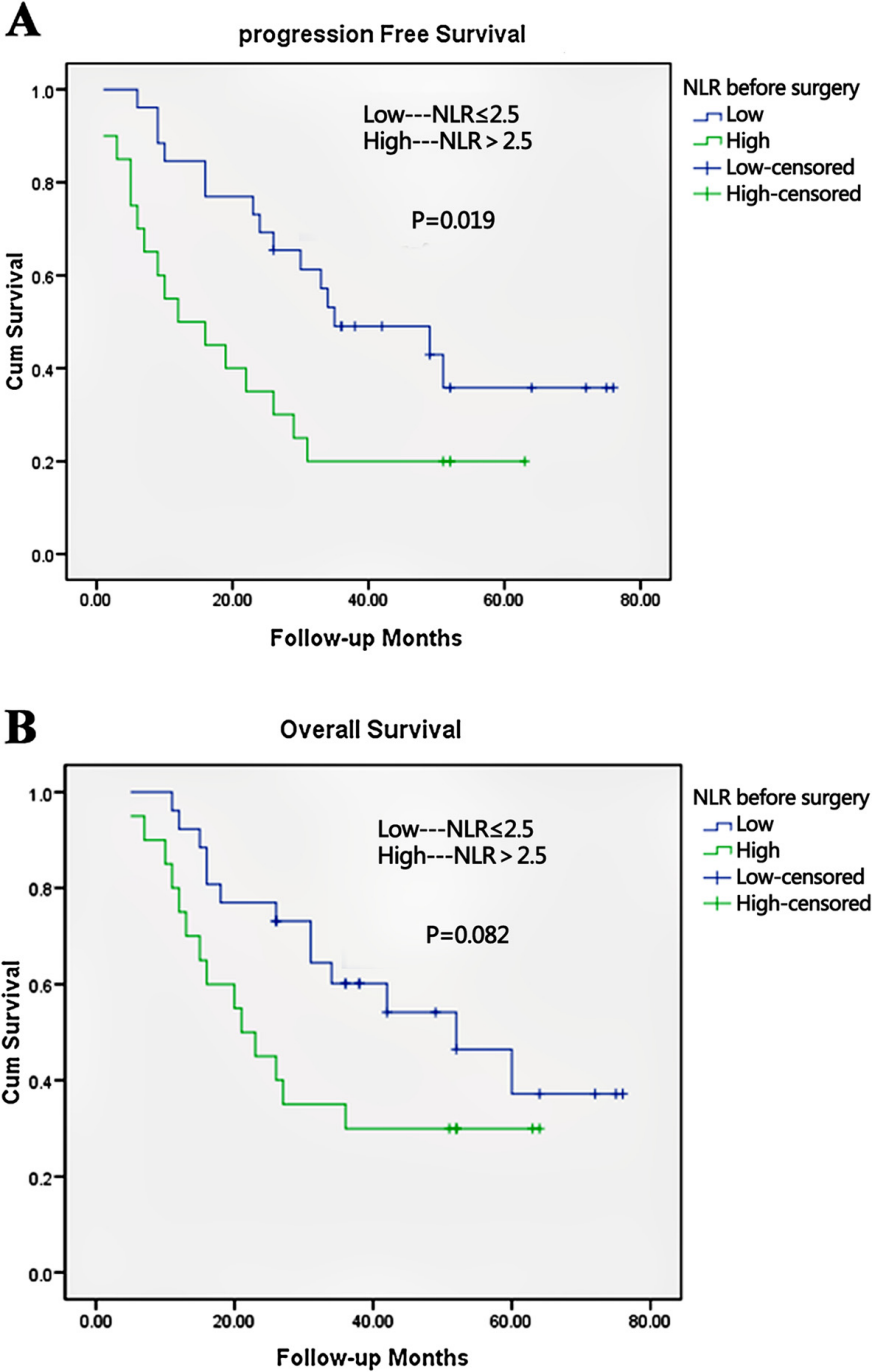
**Kaplan-Meier survival curves according to neutrophil-lymphocyte ratio (NLR) before surgery.** (**A**) Progression-free survival and (**B**) overall survival. *P* values were determined using the log-rank test.

For the 46 patients, the median PFS was 26.0 months, and the median OS was 34.0 months. In univariate analysis, variables predicting improved PFS were R0 resection, well and moderately differentiated tumor, pre-chemotherapy neutrophil count of 4,000/mm^3^ or less, pre-chemotherapy NLR 2.5 or less, pre-operative neutrophil count 4,000/mm^3^ or less, and pre-operative NLR 2.5 or less (Table 2). The following variables were associated with improved OS: R0 resection, well and moderately differentiated tumor, pre-operative neutrophil count 4,000/mm^3^ or less and pre-operative NLR 2.5 or less (Table 3). The pre-chemotherapy parameters appeared to have no prognostic values on OS (pre-chemotherapy neutrophil count *P* = 0.154; pre-chemotherapy NLR *P* = 0.113) (data not shown). Meanwhile, we found that pre-chemotherapy and pre-operative lymphocyte count had no prognostic significance using the cut-off value of 1500/mm^3^ on PFS (pre-chemotherapy lymphocytes *P* = 0.803; pre-operative lymphocytes; *P* = 0.615) or OS (pre-chemotherapy lymphocytes *P* = 0.744; pre-operative lymphocytes *P* = 0.647) in univariate analysis (data not shown). Median PFS and median OS were worse for patients with high NLR values than for those with low NLR values before chemotherapy (median PFS 16 months and 49 months, respectively, *P* = 0.012; median OS 21 months and 52 months, *P* = 0.113) (Figure 2A,B). Worse median PFS and median OS was also seen in patients with high NLR values than those with low NLR values after neoadjuvant chemotherapy but before surgery (median PFS 12 months *and* 35 months, respectively; *P* = 0.019; median OS 21 months versus 52 months; *P* = 0.082) (Figure 3A,B). Multivariate analysis identified high pre-chemotherapy NLR (*P* = 0.033, hazard ratio (HR) = 2.329, 95% CI 1.069 to 5.073) and high pre-operative NLR (*P* = 0.022, HR = 2.347, 95% CI 1.128 to 4.881) as independent factors associated with worse PFS (Table 2), but both lost independent prognostic significance for OS upon multivariate analysis (Table 3).

**Table 2:** Results of the univariate and multivariate analyses of progression-free survival in 46 patients with stage III-IV gastric cancer treated with neoadjuvant chemotherapy

**Factors**	**Number**	**Univariate analysis**^**a**^**, *P *value**	**Multivariate analysis**^**b**^**,**	
			**HR (95% CI)**	***P*value**
Radicality				
R0	37		0.163 (0.063 to 0.421)	<0.001
R1				
R2	9	<0.001	1	
Differentiation				
Differentiated^c^	15			
Poorly differentiated^d^	31	0.039		
Pre-chemotherapy blood parameters				
Neutrophils, cells/mm^3^				
≤4000	24			
>4000	22	0.065		
NLR				
≤2.5	22		1	
>2.5	24	0.012	2.329 (1.069 to 5.073)	0.033
Pre-operative blood parameters e				
Neutrophils, cells/mm^3^				
≤4000	34			
>4000	12	0.049		
NLR				
≤2.5	26		1	
>2.5	20	0.019	2.347 (1.128 to 4.881)	0.022

HR, hazard ratio; NLR, Neutrophil to lymphocyte ratio.

^a^Performed using the Kaplan-Meier analysis model and log-rank test; values of *P*<0.10 in the univariate analysis were entered into a multivariate analysis.

^b^Performed using Cox proportional hazards models with the backward likelihood method.

^c^Differentiated include: well and moderately differentiated adenocarcinoma.

^d^Poorly differentiated include: poorly differentiated adenocarcinoma, ring cell carcinoma.

**Table 3:** Results of the univariate and multivariate analyses of overall survival in 46 patients with stage III-IV gastric cancer treated with neoadjuvant chemotherapy

**Factors**	**Number**	**Univariate analysis**^**a**^**, *P *value**	**Multivariate analysis**^**b**^	
			**HR (95% CI)**	***P*value**
Radicality				
R0	37		0.127 (0.050 to 0.320)	<0.001
R1				
R2	9	<0.001	1	
Differentiation				
Differentiated^c^	15			
Poorly differentiated^d^	31	0.061		
Pre-operative blood parameters				
Neutrophils, cells/mm^3^				
≤4000	34			
>4000	12	0.037		
NLR				
≤2.5	26			
>2.5	20	0.082		

HR, hazard ratio; NLR, Neutrophil to lymphocyte ratio.

^a^Performed using the Kaplan-Meier analysis model and log-rank test; values of *P* <0.10 in the univariate analysis were entered into a multivariate analysis.

^b^Performed using Cox proportional hazards models with the backward likelihood method.

^c^Well and moderately differentiated adenocarcinoma.

^d^Poorly adenocarcinoma, ring cell carcinoma.

### Normalization of pre-chemotherapy NLR and correlation with PFS and OS

Of the 46 patients, 24 had NLR values above 2.5 before chemotherapy, and 11 of these had NLR values of 2.5 or less before surgery. Of the 22 patients with NLR values of 2.5 or less before chemotherapy, 7 had NLR values above 2.5 before surgery. The 11 patients with NLR normalization had better median PFS (35.0 versus 10.0 months; *P* = 0.003) and better median OS (60 versus 16 months; *P* = 0.042) than the 13 patients with NLR values above 2.5 before both chemotherapy and surgery, and similar median PFS (35 versus 49 months; *P* = 0.648 ) and median OS (60 versus 42 months; *P* = 0.869) as the 15 patients with NLR of 2.5 or less before both chemotherapy and surgery (Table 4).

**Table 4:** Survival of patients according to neutrophil-lymphocyte ratio (NLR) before both neoadjuvant chemotherapy and surgery

**NLR changed by chemotherapy**^**a**^			**Number**	**PFS**		**OS**	
				**Median survival, months**	***P*value**	**Median survival, months**	***P*value**
NLR >2.5	→	NLR >2.5	13	10		16	
NLR ≤2.5	→	NLR ≤2.5	15	49	<0.001	42	0.015
NLR >2.5	→	NLR ≤2.5	11	35		60	
NLR ≤2.5	→	NLR ≤2.5	15	49	0.648	42	0.869
NLR >2.5	→	NLR ≤2.5	11	35		60	
NLR >2.5	→	NLR >2.5	13	10	0.003	16	0.042

NLR, Neutrophil to lymphocyte ratio.

^a^Change between pre-chemotherapy and pre-operative NLR.

## Discussion

Gastric cancer is one of the most common types of malignancies worldwide, leading to hundreds of thousands of deaths annually. Multimodal therapy, including radiotherapy, adjuvant chemotherapy, and targeted therapy, has greatly improved the survival of patients with advanced gastric cancer. Neoadjuvant chemotherapy is currently in the limelight, with verification of its efficacy and safety now under way [29]. However, in order to select the optimal treatment regimen for individuals, accurate predictors that identify those patients who are more likely to benefit from neoadjuvant chemotherapy are needed.

Peripheral blood samples can be easily obtained for pre-treatment prediction. Several studies have established systemic inflammation-based prognostic scores before surgery [30-33]. A study conducted by Proctor *et al*. [31], which enrolled 8,759 patients diagnosed with a variety of cancers showed that the systemic inflammation-based scores, including the mGPS, NLR, PLR, Prognostic Index, and Prognostic Nutritional Index, have prognostic value. In particular, NLR has been reported to be a useful prognostic factor in gastric cancer [16-20]. Hirashima *et al*. [16] first suggested that high NLR was associated with poor OS in patients with early gastric cancer, and Jung *et al*. [20] showed that raised pre-operative NLR predicted poor disease-free survival and OS following resection for late-stage gastric cancer. However, to our knowledge, the prognostic significance of NLR in patients with advanced gastric cancer receiving neoadjuvant chemotherapy has rarely been studied. We analyzed the relationship between pre-chemotherapy and pre-operative NLR scores and survival in patients with stage III-IV gastric cancer. In addition, we evaluated whether neoadjuvant chemotherapy normalized high NLR, and evaluated the effect of such NLR normalization on survival.

Our results showed that high pre-chemotherapy NLR and high pre-operative NLR independently predicted worse PFS in patients with stage III-IV gastric cancer receiving neoadjuvant chemotherapy, and that high NLR pre-operative was associated with poor OS in univariate analysis. Although high pre-operative NLR lost its independent prognostic significance for OS in multivariate analysis, it still provided important information on NLR for clinical practice. A study by Kishi *et al.*[26] suggested that high NLR was a useful predictor of worse survival in patients with colorectal liver metastases treated with chemotherapy alone or with chemotherapy followed by hepatic resection, and our findings are consistent with this study. Kishi *et al*. also demonstrated that normalization of high NLR by chemotherapy indicated improved survival. In our study, we found that high NLR values were normalized after neoadjuvant chemotherapy in 11 patients, and that normalization of high NLR indicated improved PFS and OS, with patients in whom it occurred having similar survival to those with low NLR before both chemotherapy and surgery.

The association between elevated NLR and poor survival in patients with various types of cancers has not been clearly defined until now. It is possible that pre-treatment neutrophil and lymphocyte numbers indicate the level of inflammation within the tumor, and thus predict prognosis. Indeed, there are several possible explanations for this. Cytokines generated by neutrophils, such as vascular endothelial growth factor, interleukin-18 and matrix metalloproteinases [34-36], may establish a microenvironment that promotes angiogenesis, and thus promotes tumor growth and metastasis. In addition, the increased number of neutrophils around the tumor may suppress the anti-tumor immune responses of natural killer cells and activated T cells [37,38]. At the same time, a reduced number of lymphocytes may weaken the lymphocyte-mediated anti-tumor cellular immune response. Hence, it is likely that the combined effects of neutrophilia and lymphocytopenia lead to a high NLR and thus promote angiogenesis and inhibit anti-tumor reactivity, ultimately leading to tumor growth and progression.

To our knowledge, this is the first study of the associations of pre-chemotherapy and pre-surgery NLR values with PFS and OS in patients with advanced gastric cancer receiving neoadjuvant chemotherapy. Notably, we evaluated the prognostic significance of NLR normalization due to neoadjuvant chemotherapy for survival. Thus, the present study may provide important information for clinical practice. However, as our study was retrospective and the number of patients was small, larger numbers of patients with advanced gastric cancer treated with neoadjuvant chemotherapy should be enrolled in a well-designed prospective study. Although the peripheral blood samples were obtained 4 weeks after the last dose of chemotherapy, when hematotoxicity had been minimized, its influence could not be excluded completely. Moreover, whether the cut-off value of 2.5 for NLR is correct requires further investigation.

## Conclusions

NLR could be a convenient, easily measured prognostic indicator for patients with stage III-IV gastric cancer treated with neoadjuvant chemotherapy. Patients with high pre-treatment NLR values need multimodal therapy, and normalization of high NLR by neoadjuvant chemotherapy indicates a good chemotherapy response rate and improved survival. Pre-treatment NLR may help clinicians to identify those patients who will benefit from neoadjuvant chemotherapy, and normalization of NLR by chemotherapy may represent a good prognostic indicator. However, further studies involving greater numbers of patients with gastric cancer are required.

## Abbreviations

AJCC: American joint of committee on cancer; CR: Complete response; CRP: C-reactive protein; FOLFOX: Oxaliplatin plus 5-fluorouracil plus leucovorin; HR: Hazard ratio; mGPS: Modified glasgow prognostic score; NLR: Neutrophil-lymphocyte ratio; OS: Overall survival; PLR: Platelet-lymphocyte ratio; PFS: Progression-free survival; PD: Progressive disease; PR: Partial response; SD: Stable disease; Tis: Tumor in situ; TNM: Tumor, node, metastasis; XELOX: Oxaliplatin plus capecitabine.

## Competing interests

The authors declare that they have no competing interests.

## Authors’ contributions

HLJ and GEZ conceived of the study, collected data, performed analysis, and drafted the manuscript. XKL and CC participated in literature search and coordination. HY and XMH performed the statistical analysis. XSL and QZ participated in the treatment of the patients. JRY participated in study design and helped to draft the manuscript. All authors read and approved the final manuscript.
